# Crush syndrome in a case of severe infant physical abuse: a case report

**DOI:** 10.11604/pamj.2021.39.172.30309

**Published:** 2021-07-06

**Authors:** Maria Florou, Vassilis Lambropoulos, Vasileios Mouravas, Chrysostomos Kepertis, Dimitrios Godosis, Kleanthis Anastasiadis, Christos Kaselas, Savvas Mavromatidis, Ioannis Spyridakis

**Affiliations:** 1 Second Paediatric Surgery Department, Aristotle University of Thessaloniki, “Papageorgiou” General Hospital, Thessaloniki, Greece,; 2 Operating Theaters, Surgical Sector, “Papageorgiou” General Hospital, Thessaloniki, Greece

**Keywords:** Rhabdomyolysis, crush syndrome, child, infant, case report

## Abstract

Crush syndrome, also known as traumatic rhabdomyolysis, is the result of the disruption of skeletal muscle fibers with the release of intracellular contents into the bloodstream. Although trauma is the main trigger for rhabdomyolysis in adults, in the pediatric population viral infections and inherited disorders seem to be the most frequent causes. Only a few reports in the literature mention rhabdomyolysis secondary to non-accidental pediatric trauma. We herein report an unusual case of traumatic rhabdomyolysis, following significant physical abuse in an infant. Rhabdomyolysis should be suspected in children presenting with a history of excessive blunt trauma, because a prompt diagnosis and treatment prevent from the potential life-threatening consequences.

## Introduction

Pediatric rhabdomyolysis is a clinical condition that accounts for approximately 5% of total cases of acute kidney injury cases in the pediatric age group [1]. It results from damaged striated muscle fibers and the leakage of sarcoplasmic substances, such as myoglobin, creatine kinase, potassium and phosphorus, into the circulation. The diagnosis is based on meticulous history intake and clinical suspicion and is confirmed by the elevated levels of plasma creatine kinase and the urine myoglobin. A wide range of conditions may result in rhabdomyolysis, with infections and genetic disorders being the most common [2]. Here we describe a rare case of traumatic rhabdomyolysis, synonymous to crush syndrome, associated with severe physical abuse in an infant.

## Patient and observation

**Patient information:** an eighteen-month-old male presented to our emergency department with apparent signs of physical abuse, while vomiting. He had a low level of consciousness and he was responsive only to painful stimuli when the alert, verbal, pain, unresponsive (AVPU) scale was applied. He was brought by his mother and a few hours prior to presentation the boy had suffered excessive physical abuse by his mother´s partner, who was a drug addict. The child´s past medical and surgical history were uneventful.

**Figure 1:**
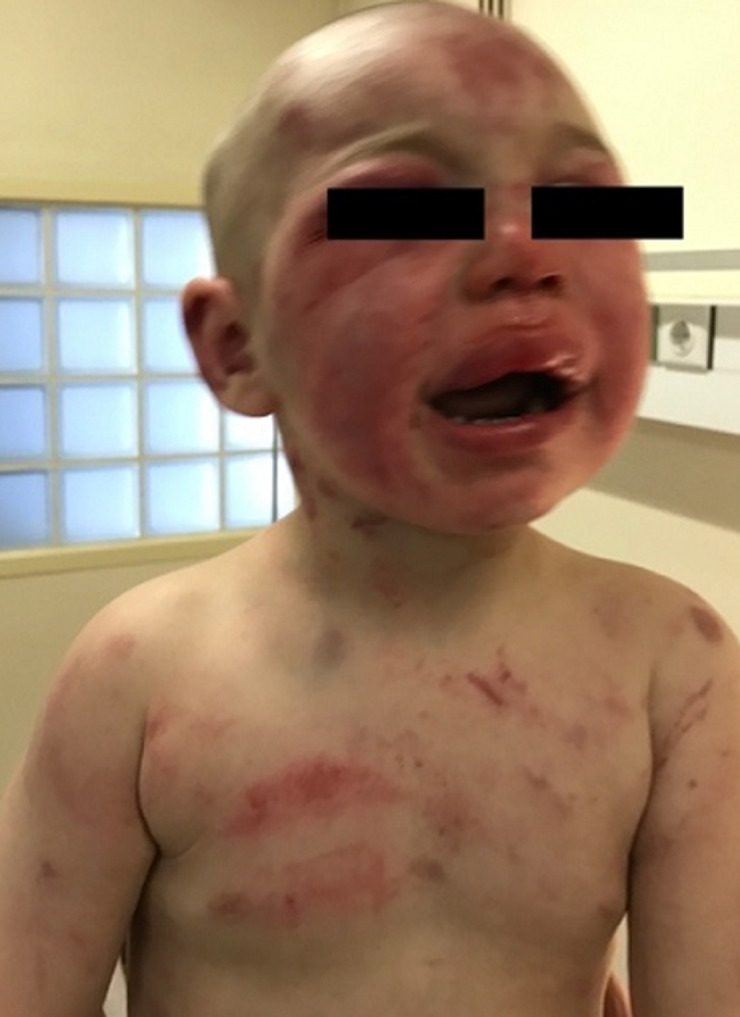
multiple bruising on face, head and chest

**Figure 2:**
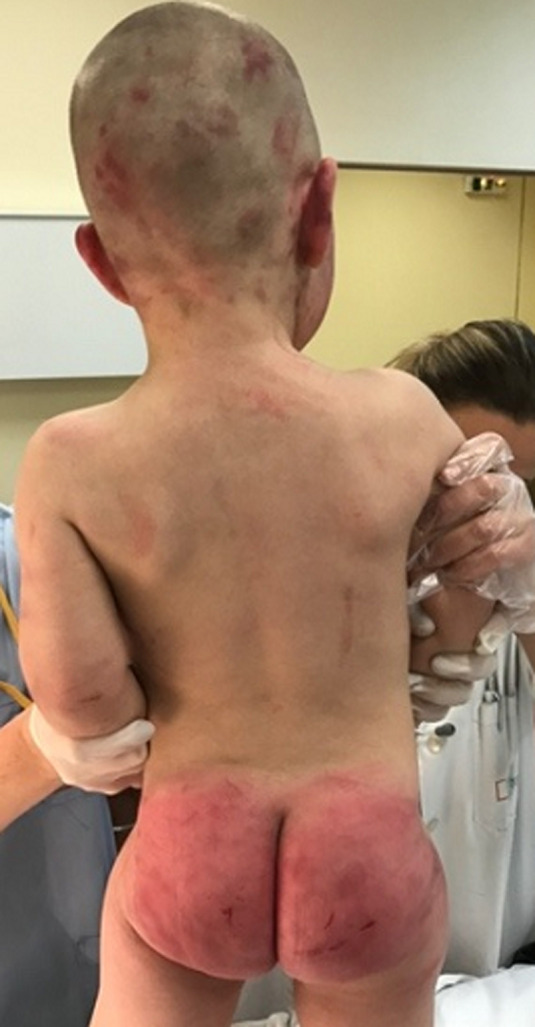
ecchymoses on buttocks

**Clinical findings:** physical examination revealed injuries all over his small body. Multiple bruising and edemawere noted on his face and his head (Figure 1). Extensive ecchymoses and soft tissue swelling extended over his buttocks (Figure 2). His chest, upper and lower limbs were covered with bruises, scratches and cigarette burns. Many skin lesions and burn scars were on different stages of the healing process, both evident that the child had been repeatedly beaten in the past.

**Diagnostic assessment:** skeletal surveys did not reveal any fractures and the computed tomography of the brain was normal. Both kidneys and the intraabdominal organs had normal appearance in the ultrasound examination. A complete laboratory examination was applied, and here we mention some of the results; white blood count (WBC) 22.70 x 10^9^/L, BUN 55mg/dL, creatinine 0.66 mg/dL, lactate dehydrogenase (LDH) 1133 U/I, creatine kinase 1209 U/I. The urine analysis showed; 1.031 specific gravity, protein 500 mg/dl, glucose 300 mg/dL, ketone 15 mg/dL and heme 3+.

**Diagnosis:** after the completion of the tests, the diagnosis of excessive blunt trauma with subsequent onset of crush syndrome was made.

**Therapeutic intervention:** the boy was admitted for intravenous fluid resuscitation, clinical monitoring and more laboratory examinations. He was hemodynamically stable and retained a good urine output from the first day of hospitalization. On the second day, the creatine kinase peaked at 3172 U/I and then declined steadily, along with improvement of his clinical condition.

**Follow up and outcomes:** after 9 days of hospitalization, he was discharged and social workers took care of the child.

**Patient´s perspective:** during the time he was hospitalized the child was scared and anxious. The first two days it was hard for him to sleep at night, but the nurses and the social workers made him feel comfortable along with his clinical improvement.

**Informed consent:** the patient´s mother was informed about the case report and the reasons why this incident was interesting for the authors. She gave informed consent allowing the authors to use the boy´s story and picture.

## Discussion

Rhabdomyolysis is a common clinical condition in the pediatric population and is responsible for approximately 5% of the total cases of acute kidney injury [1]. It is the systemic manifestation of the damaged striated muscle fibers and the subsequent release of the sarcoplasmic elements into the plasma. There is a wide range of etiologies and the most frequently seen are: infections, genetic syndromes, congenital disorders and medications [2]. Traumatic rhabdomyolysis, also known as crush syndrome, is a rare occurrence and results from excessive soft tissue trauma caused by natural disasters, motor vehicle accidents, entrapments, prolonged exercise and physical abuse [3]. Rhabdomyolysis, whether traumatic or not, has a typical triad of symptoms; myalgia, weakness and dark, tea-colored urine [4]. A detailed history intake and clinical suspicion are the key of a prompt diagnosis.

The confirmation of rhabdomyolysis is based on the serum levels of creatine kinase; greater than 1,000 U/L or greater than 5 times the upper normal limit. Creatine kinase is the diagnostic marker for muscle damage and has no adverse effects on the kidneys. Its serum levels will peak 2 to 3 days post injury and then drop by one half every 48 hours [5]. The myoglobin and the heme have direct and indirect nephrotoxic effects and are responsible for the acute kidney failure, secondary to rhabdomyolysis. Early therapeutic measures should be applied, to prevent the complications of rhabdomyolysis, including: electrolytes imbalances, acute renal failure, compartment syndrome, cardiac arrythmia and disseminated intravascular coagulation [6]. The management is supportive and includes aggressive fluid resuscitation, monitoring, correction of the electrolyte abnormalities and avoidance of nephrotoxic medications. The use of bicarbonate and mannitol is applied on adults, but there is not enough evidence in the literature for the children. There is also a role for hemodialysis in patients with oliguric renal failure and hyperkalemia, but is relatively rare [2,7]. Our patient, retained a good urine output and the levels of creatinine were within the normal limits during its hospitalization. Fortunately, children keep a lower risk of acute renal failure from rhabdomyolysis than the adults [3,8].

The discharge criteria are based on clinical improvement, normal levels of electrolytes, creatine kinase and creatinine and a good urine output. The patients should be followed up by their pediatrician [1].

## Conclusion

Rhabdomyolysis should be considered when children present in the emergency room with a history of blunt trauma and extensive bruising on their bodies. The acute onset of this condition and its potential life-threatening consequences, urge a high clinical suspicion, for a prompt diagnosis and treatment.
